# Determination of the Geometric Parameters of Electrode Systems for Electrical Impedance Myography: A Preliminary Study

**DOI:** 10.3390/s22010097

**Published:** 2021-12-24

**Authors:** Andrey Briko, Vladislava Kapravchuk, Alexander Kobelev, Alexey Tikhomirov, Ahmad Hammoud, Mugeb Al-Harosh, Steffen Leonhardt, Chuong Ngo, Yury Gulyaev, Sergey Shchukin

**Affiliations:** 1 Department of Medical and Technical Information Technology, Bauman Moscow State Technical University, 105005 Moscow, Russia; 9784882@mail.ru (V.K.); ak.mail.ru@gmail.com (A.K.); tikhomirov.an@bmstu.ru (A.T.); Hammoud@bmstu.ru (A.H.); alharosh@bmstu.ru (M.A.-H.); schookin@bmstu.ru (S.S.); 2 Chair of Medical Information Technology, RWTH Aachen University, 52074 Aachen, Germany; leonhardt@hia.rwth-aachen.de (S.L.); ngo@hia.rwth-aachen.de (C.N.); 3 Kotelnikov Institute of Radioengineering and Electronics (IRE) of Russian Academy of Sciences, 125009 Moscow, Russia; gulyaev@cplire.ru

**Keywords:** electrical impedance, electrode system, Pareto optimality, physical modeling, mathematical model, MRI reconstruction, neuromuscular interface, bionic control, prosthesis, orthoses

## Abstract

The electrical impedance myography method is widely used in solving bionic control problems and consists of assessing the change in the electrical impedance magnitude during muscle contraction in real time. However, the choice of electrode systems sizes is not always properly considered when using the electrical impedance myography method in the existing approaches, which is important in terms of electrical impedance signal expressiveness and reproducibility. The article is devoted to the determination of acceptable sizes for the electrode systems for electrical impedance myography using the Pareto optimality assessment method and the electrical impedance signals formation model of the forearm area, taking into account the change in the electrophysical and geometric parameters of the skin and fat layer and muscle groups when performing actions with a hand. Numerical finite element simulation using anthropometric models of the forearm obtained by volunteers’ MRI 3D reconstructions was performed to determine a sufficient degree of the forearm anatomical features detailing in terms of the measured electrical impedance. For the mathematical description of electrical impedance relationships, a forearm two-layer model, represented by the skin-fat layer and muscles, was reasonably chosen, which adequately describes the change in electrical impedance when performing hand actions. Using this model, for the first time, an approach that can be used to determine the acceptable sizes of electrode systems for different parts of the body individually was proposed.

## 1. Introduction

Determining the type and parameters of the performed action is a device control systems key issue, which is based on bionic control. Such devices can be both medical devices (bioelectric prostheses, orthoses, exoskeletons, rehabilitation, surgical devices) and special-purpose devices.

The problems of developing an anthropomorphic [1] neuromuscular interface have been considered in many studies [2,3,4]. The key point in the neuromuscular interfaces’ development is the definition of methods and biophysical principles for obtaining information about the performed movement, which should allow the determination of not only its type but also to carry out a numerical assessment of the movement force-torque characteristics [1,5].

Management based on surface electromyography is considered a classic. The method allows the non-invasive recording of the skin’s potential arising from muscle excitation. Analysis of a signal’s amplitude-frequency characteristics allows realizing discrete [6], proportional [6], and pattern recognition-based control [7]. Alternative approaches are proposed to expand the functionality and overcome the typical limitations of this method [7]. One of the methods is based on the electrical impedance (EI) analysis [8,9,10].

EI studies are based on the passing a high-frequency probing current principle (with a frequency of 50–100 kHz and an amplitude of 1–10 mA) between current electrodes (CE) of the electrode system (ES) and recording the arising potential difference at the potential electrodes (PE) when they are located under the tetrapolar lead system [10,11,12] (CE at the edges, PE in the middle) in the projection of the range of interest [1]. The measured EI values carry information about the electrical biological tissues’ properties at the probing depths. EI studies are used in the application for predicting or detecting (diagnosing) several diseases of the neuromuscular apparatus [13,14], assessing the blood supply to organs, for detecting peripheral veins [15] and venepuncture [16], for evaluating and adjusting treatment [17] or rehabilitation process [9].

In this work, EI myography, which can be used to solve bionic control problems, which consists in assessing the change in the EI value during muscle contraction in real-time is considered [1]. It is one of the earliest EI research [18,19] applications that has not lost its relevance today [1,10]. Despite the existing contradictory results obtained during attempts “ex vivo” [18,19] and “in vivo” [8,20], EI changes during isometric contractions and tetanic stimulation assessments, the concept of using EI for bionic control is attractive since it can provide new knowledge about biophysical processes occurring in muscle and surrounding tissues associated with the EI myography signals parameters formation.

**Figure 1:**
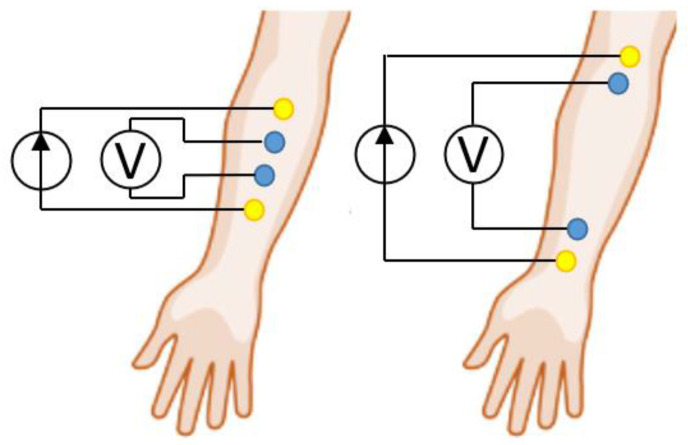
An example of the ES size and location for upper limb EI myography.

The location of the ES in the EI of myography is important from the amplitude characteristics of the signal and reproducibility point of view when conducting experimental studies. So, in existing studies, as a rule, ES are used with electrodes located along the axis of the studied muscle groups [21]. The performed literature review shows that the existing approaches do not always take into account the need to select an acceptable ES size (distance between electrodes) in EI myography, which is important in terms of the EI signal expressiveness and reproducibility [8,9,10,13]. Figure 1 shows that the individual mechanisms contribution for the EI signal conditioning described above may differ depending on the ES sizes, which sometimes leads to obtaining different results of EI studies. So, for example, at large interelectrode distances, the EI signal conditioning is mainly associated with the muscle’s resistance change and blood filling, and with an ES size comparable to the limb characteristic dimensions, with a change in the skin-fat layer thickness [22].

In this work, we consider two main contributions that affect EI signal conditioning when the small size ES (Figure 1, left) is located in the muscle projection area or forearm muscle group, which are active during the motion: a change in the skin-fat layer thickness and muscle resistivity. None of these signal conditioning mechanisms can be achieved separately, which is difficult in terms of evaluating the above contributions in practice [20], as a result, in the present work, attempts to use simulation were made.

**Figure 2:**
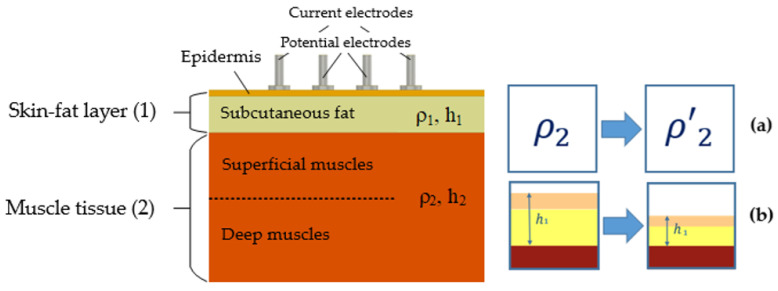
EI myography conditioning mechanisms: (**a**) change in muscle resistivity; (**b**) changes in skin-fat layer thickness (h_1_ is the skin-fat layer thickness; h_2_ is the muscle tissues thickness; ρ_1_ is the skin-fat layer electrical resistivity, ρ_2_ is the muscle tissues electrical resistivity).

In the contractions process, the muscle body thickens, the skin-fat layer becomes thinner due to deformation from the pressed ES (Figure 2a), and the muscle mutual position and the epicutaneous ES is changed [20,23]. Considering the muscles electrical properties [10,24,25,26] and their change during contraction (Figure 2b) [18,19], including spatial dependence [27,28], during the action, a current density redistribution in the measurement area occurs [23]. Everything noted is more related to superficial muscles; however, in the case of deep muscles consideration, which are covered by superficial ones on the forearm, then during their contraction, passive deformation of the latter occurs, which also affects the amplitude parameters of the EI measured and can lead to the interpretation of measurement results for superficial and deep muscles being different.

Thus, this work is devoted to determining the acceptable ES size for EI myography using the Pareto-optimality assessment method and the model for the EI signals conditioning in the forearm area, taking into account the change in the skin-fat layer and muscle groups electrophysical and geometric parameters when the brush acting. This study consists of two parts, including a preparatory part and a main part. In a preparatory part, using finite element modeling, an acceptable detailing of the investigated area of the forearm real geometric model was substantiated in terms of the absolute values of electrical impedance for different sizes of electrode systems. The results obtained made it possible to reasonably use the mathematical model when assessing the geometric dimensions of electrode systems for electrical impedance myography; this methodology is described in the main part.

## 2. An Acceptable Model of Electrical Impedance Myography Based on Numerical Simulation Determination

To achieve theoretical EI studies when changing the skin-fat layer and muscle groups’ electrophysical and geometric parameters, it is necessary to establish a model that allows establishing the electric field intensity in the range of interest. Numerical simulation was performed to determine a sufficient degree of forearm anatomical features detailing in terms of the EI measured and to substantiate the possibility of using forearm analytical EI models during muscle contraction. The imported forearm models were forearm anatomical models with different simplification levels.

### 2.1. Software

**Figure 3:**
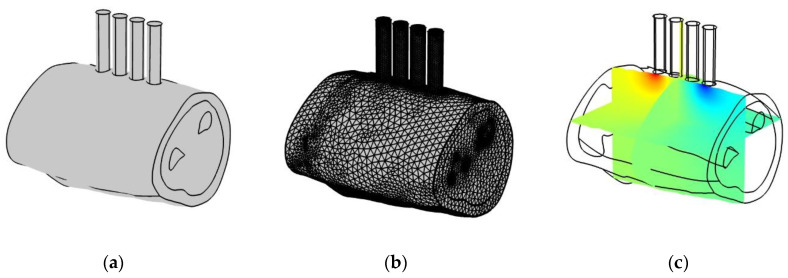
Potential distribution in the forearm anatomical model simulation by the finite element method: (**a**) geometric representation of the model; (**b**) discretization of the model into geometric primitives; (**c**) potential distribution.

The calculations were performed using the COMSOL Multiphysics 5.4 software package. The package interfaces use the finite element method to solve the components of partial differential equations [29]. Figure 3 shows that the main advantage of this method is its adaptability to complex geometry and, including for the imported forearm model [10,30,31], the ability to calculate the current density and equipotential surfaces at any point in the model.

**Figure 4:**
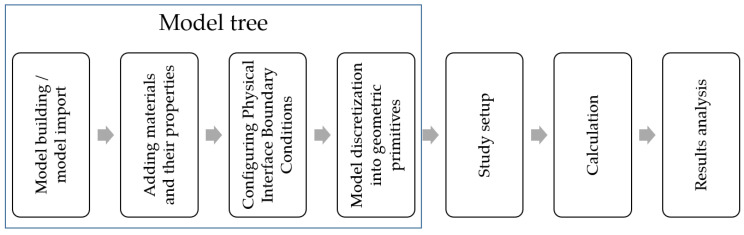
Research algorithm in COMSOL Multiphysics 5.4.

To calculate the electric field distribution, current, and potential in a conductive medium of three-dimensional space, the physical interface “Electric currents” AC/DC was used, which implements the solution of the Maxwell system of equations when dividing into finite elements: a grid that is a mosaic of tetrahedrons. Figure 4 shows the research algorithm diagram for performing in the program.

### 2.2. Volunteer MRI Forearm Anatomical Models

A 3D reconstruction of seven volunteers’ forearms MRI images was performed to obtain forearm anthropometric models. The seven volunteers included four men and three women aged 18–35 years, with a girth in the upper third of the forearm from 0.2 to 0.35 m, without diagnosed pathologies of the upper limbs.

**Figure 5:**
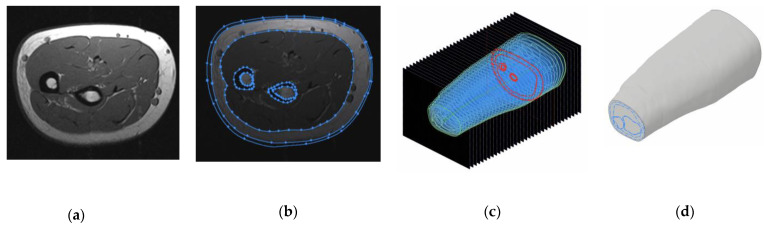
Volunteers’ forearms MRI images reconstruction: (**a**) MRI slice; (**b**) contouring of the slice; (**c**) contoured slices series; (**d**) forearm 3D reconstruction.

Figure 5a shows that the reconstruction was carried out as follows: the obtained series of MRI images were loaded into the 3D design system Autodesk Inventor. Figure 5b shows MRI processing by manual contouring using the “Spline” function allowed to conditionally represent a model of probing environment as multilayer, consisting of a skin-fat layer, muscle tissue, and bone tissue. Figure 5c shows that the result of this procedure is a series of contoured equidistant sections obtained by the MRI study parameters. Figure 5d shows using the built-in “Loft” function that forearms anatomical 3D models were built based on the series of contours.

### 2.3. Forearm Models Simplification

**Figure 6:**
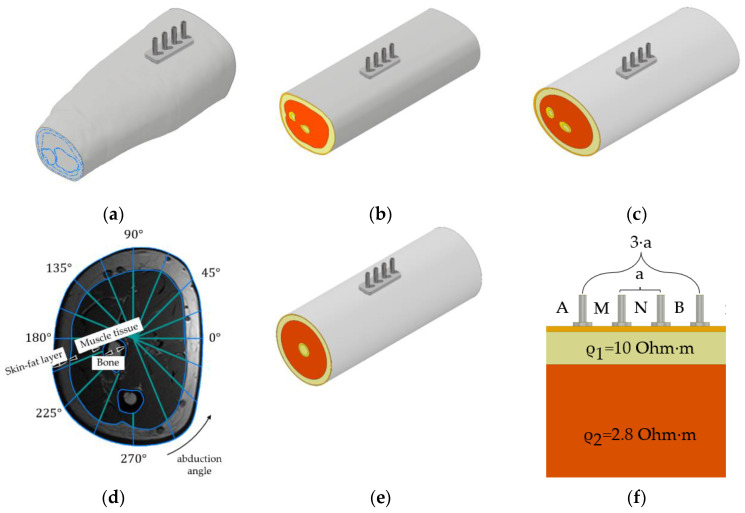
Model simplification error investigation: (**a**) anatomical; (**b**) single cut; (**c**) ellipsoidal; (**d**) one-dimensional forearm MRI sections; (**e**) cylindrical (for 16 slices); (**f**) planar.

Figure 6a shows that the anatomical models’ simplification was performed manually to single-cut (Figure 6b), ellipsoidal (Figure 6c), one-dimensional (Figure 6d), and cylindrical models (Figure 6e). A single-cut model was obtained as a result of the operation “extrusion” of an MRI slice of the forearm in the upper third; an ellipsoidal model was based on the approximation of the media contours by the least-squares method to ellipses. Figure 6f shows that the cylindrical and planar models’ thicknesses media were calculated relative to one-dimensional sections of the MRI slice for 16 positions of the ES.

### 2.4. Model Parameters and Assumptions

All models were presented as an isotropic multilayer with resistivity close to the skin-fat layer resistance, muscle tissue, and bone tissue at a frequency of 100 kHz [32,33,34]: 10 Ohm·m, 2.8 Ohm·m, and 100 Ohm·m, respectively. Since the calculation was carried out at the same frequency with the known parameters of the mean for it, in COMSOL Multiphysics, a stationary investigation in which electromagnetic fields do not change over time was used. Thus, since the EI values were simulated at one frequency, at which the effect of the capacitive component is an order of magnitude less than the active component of the impedance, and the electrical parameters of the media corresponded to it, the dependence of the capacitive component of the medium on frequency was not considered [35]. That is, the EI results were presented as for impedance without separation into active and responsive components. If necessary, this assumption can be adapted considering the frequency parameter and, thus, it is possible to consider ideal and anomalous capacitive effects, which were estimated using classical EI measurements [36,37].

A 1 mA current was supplied through the CE (to A with a positive sign, to B with a negative sign), and the potential difference across the PE was calculated (M and N, respectively). The EI value was calculated by dividing the calculated potential difference by the current value.

In the case of the anatomical model, the ES was located in the upper third of the forearm in a typical installation site for existing bioelectric devices. The ES was located in the middle for the simplified models due to their symmetry relative to the longitudinal axis, and the models’ size was chosen to be eight times larger than the interelectrode distance by Appendix A. For the model discretization into geometric primitives, a mesh with an evenly increasing division with the parameters presented in Table A1 Appendix B was used.

The electrodes were assumed to be point-like, and, as a consequence, factors such as the electrode shape, the “electrode-skin” contact area, and edge effects were not taken into account [12]. From the electric impedance value point of view, the possibility of using this approach was investigated and substantiated in a previous paper [38]. In order to unify the design and reduce the number of ES-specified geometric parameters, the distance between the electrodes is taken to be equidistant from each other at a distance a, which is in accordance with the Wenner tetrapolar system (1) [39].

AM = MN = NB = a(1)

### 2.5. Finite Element Simulation Results

**Figure 7:**
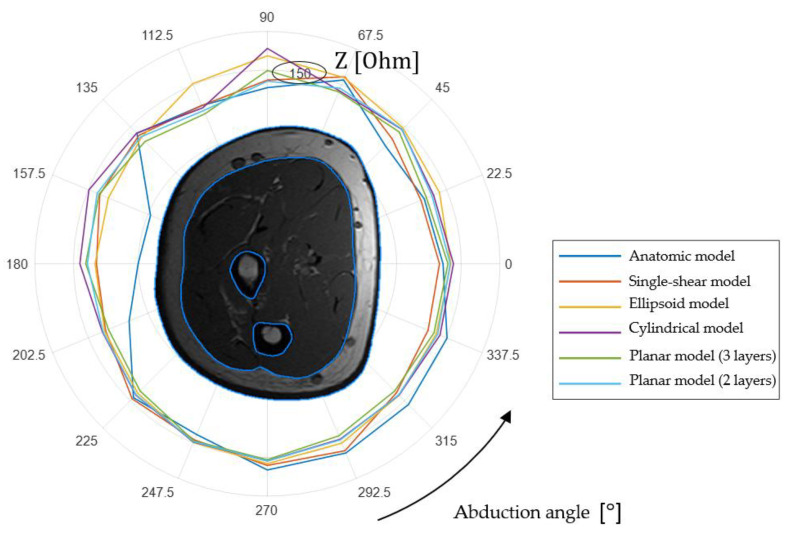
EI simulation results for anatomical and simplified models (Z is an absolute EI value).

Using the models obtained by finite element simulation, the EI values for different sizes point ES were found, and the models’ simplification error was estimated. Data processing and analysis were carried out using the MATLAB R2020b programming and numeric computing platform. Figure 7 shows the polar diagram, which is an example of the EI values simulated for one of the volunteers’ forearm models considered (with a forearm girth of 0.35 m; 16 slices) at ES different positions.

Table 1 shows the absolute S values and simplified models relative S/Z RMS EI obtained by simulation relative to the anatomical (2) for the presented volunteer (Figure 7), which was calculated for ES different locations.

(2) $$S=\sqrt{\frac{\sum_{i=1}^{N_{\mathrm{slices}}}{\left(Z_{i}\;{-\;Z}_{\mathrm{anat}}\right)}^{2}}{N_{\mathrm{slices}}}}$$

According to the analysis, the two-layer model gives good results when using small size ES in comparison with the limb characteristic radius. However, when approximating the forearm study area with a planar model, it is necessary to take into account that with an increase in the interelectrode distance, the effective probing depth increases. Since the forearm geometry is more similar to a cylinder, the error in the approach of using a planar model will increase with the ES size increase. Appendix C shows the results of numerical simulation for determining the ES range for which the representation of the forearm studied area can be simplified as a planar model.

Thus, the simulation results substantiate the use of a two-layer model, which is represented by the skin-fat layer and muscles. This model allows to adequately calculate the EI values provided that the ES size does not exceed the limb characteristic dimensions (the limb characteristic radius does not exceed the interelectrode distance by three times). In addition, this model allows doing the analytical calculations with a minimum number of specified geometric parameters. The advantage of using analytical approaches is the possibility to obtain a mathematical expression for the relationship between the measured EI and all geometric and electrical parameters of the media that characterize this model. This approach is used in further research.

## 3. Methodology for Selecting ES Geometric Dimensions for EI Myography

**Figure 8:**
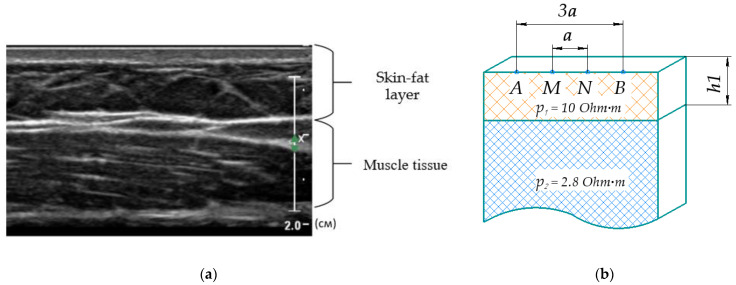
(**a**) Longitudinal forearm ultrasound investigation; (**b**) a homogeneous half-space two-layer model, represented by the skin-fat layer and muscle tissues, A and B—CE, M and N—PE; ρ_1_ is the skin-fat layer electrical resistance, ρ_2_ is the muscle tissues electrical resistivity, h1 is the skin-fat layer thickness.

An important task in the control systems for technical devices based on EI design is to determine the ES geometric parameters. This ES provides the necessary measured EI signals sensitivity to determine the type of action performed [10]. Figure 8 shows that on the forearm surface, a two-layer model justified above with the first layer thickness was used to solve this problem and calculate the EI (corresponding to the operator skin-fat layer), which can be determined individually based on the longitudinal forearm ultrasound investigation.

Papers [12,39] show an analytical expression that was obtained for the relationship between the EI for point electrodes (3) and model parameters such as the interelectrode distance a; AB, the distance between the current electrodes (3a for the considered ES); MN, the distance between the potential electrodes (a for the considered ES); the skin-fat layer ρ_1_ and muscle tissues ρ_2_ electrical resistances; and the skin-fat layer thickness h_1_ for this model. Verification of the possibility of using an analytical relationship of electrical impedance for a two-layer model was performed using finite element modeling similar to Appendix A.

(3) $$Z=\frac{{2\rho }_{1}}{\pi }(\frac{1}{\mathrm{AB}-\mathrm{MN}}-\frac{1}{\mathrm{AB}+\mathrm{MN}}+2\overset{\infty }{\underset{n=1}{\sum }}{\left(\frac{\rho_{2}-\rho_{1}}{\rho_{2}{+\rho }_{1}}\right)}^{n}\cdot \left[\frac{1}{\sqrt{{\left(\mathrm{AB}-\mathrm{MN}\right)}^{2}+{\left({4\mathrm{nh}}_{1}\right)}^{2}}}-\frac{1}{\sqrt{{\left(\mathrm{AB}+\mathrm{MN}\right)}^{2}+{\left({4\mathrm{nh}}_{1}\right)}^{2}}}\right])$$

The skin-fat layer thickness effect on the EI value can be demonstrated based on the apparent resistance (AR) calculation (4).

(4) $$\rho_{a}=2\pi \alpha Z$$

The effective ES probing depth ceases to capture the muscle tissue layer, and the AR increases as the ratio of the interelectrode distance to the skin-fat layer thickness $a/h_{1}$ decreases. This means that the contribution to the change in the EI signal associated with the change in the muscle resistivity during contraction becomes smaller.

(5) δρa(ah1)=ρ1−ρa(ah1)ρ1−ρ2

**Figure 9:**
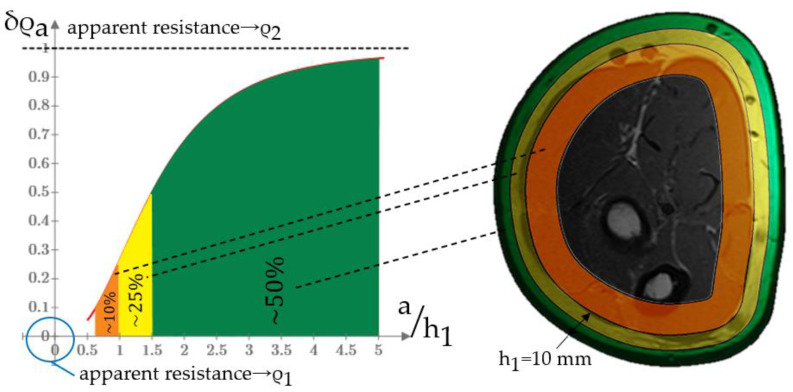
Relationship between the AR relative change and the ratio of the first layer thickness to the interelectrode distance (**left**) and its projection on the volunteer’s forearm MRI (girth 0.35 m) for ES with an interelectrode distance 0.01 m (**right**).

Figure 9 shows the AR relative change graph projection using the volunteer MRI section example with a forearm girth of 0.35 m and a skin-fat layer thickness of 0.01 m. In the case of using ES with an interelectrode distance of 0.01 m, the change in muscle resistivity during contraction will make a relative change in EI of no more than 25%. To increase this value, it is necessary to increase the interelectrode distance, which is limited by the requirements for the ES design and the measurement method. Taking these factors into account, it is necessary to use an individually-oriented approach to determine an acceptable interelectrode distance, which makes it possible to measure the muscle contraction parameters with higher sensitivity.

### 3.1. Acceptable ES Sizes Criteria

Solving the problem of determining the acceptable ES size for EI myography based on the papers [12,40] analysis and literature review [18,19,41,42], it is known that during contraction, the muscle electrical resistivity changes. It is possible to estimate the skin-fat layer thickness and its change in the process action using dynamic ultrasound and MRI investigation. Based on the studies carried out related to the assessment of morphological changes in the forearm, it was found that the thickness of the skin-fat layer becomes thinner in the process of performing the action. Considering that the electrical conductivity of muscles is several times higher than that of the skin-fat layer, it is expected that the apparent resistance will decrease with its thinning, and, accordingly, the EI will decrease, too.

Thus, four optimality criteria for choosing the ES size depending on the skin-fat layer thickness and electrical resistance were determined. So, for EI myography from the subsequent interpretation of signals point of view, it is important to determine the absolute and relative changes in signals as a result of muscle activity. In case of an analytical expression of EI, these changes can be represented as the values of the EI derivative for the studied parameter for the thickness of the skin-fat layer and the conductivity of muscle tissue, respectively. That is why the criteria were absolute (${\mathrm{dZ}}_{{\mathrm{dh}}_{1}}$, ${\mathrm{dZ}}_{{d\rho }_{2}}$) and relative (${\mathrm{dZ}}_{{\mathrm{dh}}_{1}}/Z$, ${\mathrm{dZ}}_{{d\rho }_{2}}/Z$) relationships between the partial derivatives of a two-layer model EI expressed by the skin-fat layer thickness (6) and the muscle tissues resistivity, respectively (7).

(6) dZdh1=4ρ1π∑n=1∞(ρ2− ρ1ρ2+ρ1)n(16n2h1((AB+MN)2+16n2h12)32-16n2h1((AB- MN)2+16n2h12)32)

(7) $${\mathrm{dZ}}_{{d\rho }_{2}}=\frac{4\cdot \rho_{1}}{\pi }\overset{\infty }{\underset{n=1}{\sum }}n(\frac{1}{\sqrt{{\left(\mathrm{AB}\;-\;\mathrm{MN}\right)}^{2}+{\left({4\mathrm{nh}}_{1}\right)}^{2}}}-\frac{1}{\sqrt{{\left(\mathrm{AB}+\mathrm{MN}\right)}^{2}+{\left({4\mathrm{nh}}_{1}\right)}^{2}}})\left(\frac{1}{\rho_{1}{+\rho }_{2}}-\frac{\rho_{2}-{\;\rho }_{1}}{{\left(\rho_{1}{+\rho }_{2}\right)}^{2}}\right){\left(\frac{\rho_{2}-{\;\rho }_{1}}{\rho_{1}{+\rho }_{2}}\right)}^{n-1}$$

**Figure 10:**
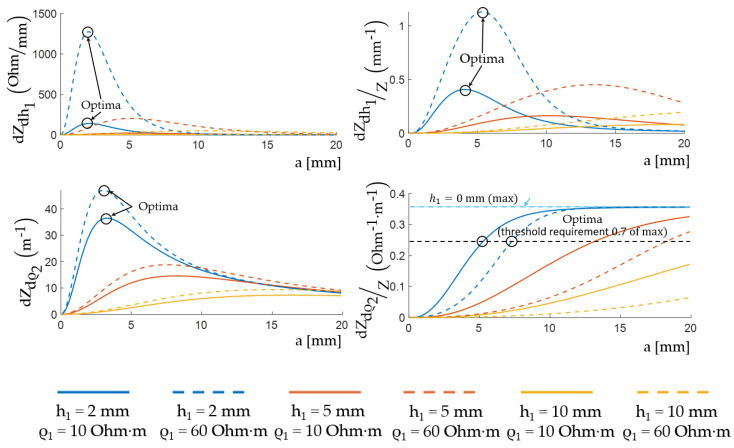
Relationship between the two-layer model EI expression partial derivatives and the interelectrode distance.

Figure 10 shows the EI change sensitivity depending on the thickness and skin-fat layer resistivity using analytical expressions for the optimality criteria and the MATLAB R2020b according to the assessment done. The relationsips were constructed for the range of thicknesses of the skin-fat layer from 2 to 10 mm and different specific electrical resistances of the skin-fat layer from 10 to 60 Ohm·m, which are considered acceptable for a frequency of 100 kHz [32,33,34]. The range of values for the thickness of the skin-fat layer was selected based on the individual characteristic sizes of volunteers MRI and previous studies [43]. If the ES size is small, the effective probing depth covers only the skin-fat layer, and if it is large enough, the probing depth corresponds to the muscle layer. The curves peaks correspond to the acceptable ES size, at which the ES is sensitive to changes in the medium parameters and the maximum change in the signal is observed for each presented criterion. The 70% threshold of the maximum value was chosen as the optimum for the relative change in the muscle tissues electrical resistivity criterion due to the ES design limitations.

**Figure 11:**
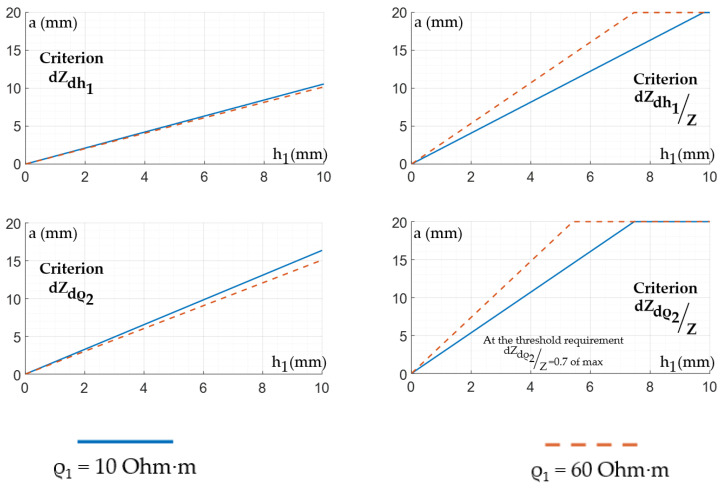
Relationship between the interelectrode distance values and the skin-fat layer thickness.

Figure 11 shows the diagrams for selecting the sizes of the ES depending on the thickness and conductivity of the skin-fat layer, which were obtained for acceptable sizes. According to these relationships, it can be seen that the size of the ES weakly depends on the resistivity of the skin-fat layer, and it much more strongly depends on its thickness.

### 3.2. ES Size Selection by Pareto Optimality

To formalize the optimality problem, objective functions depending on variables with unknown values, which were varied in the optimization process to obtain an optimal solution, are used. When one optimality criterion is considered, the search usually boils down to obtaining the largest, as in the present case, or the smallest value of this criterion, that is, to solve the problems of maximization or minimization.

Since the EI change during muscle contraction is determined both by a change in the skin-fat layer thickness and by a change in the muscle tissue conductivity, the ES optimal sizes for these two processes are different. In the case when not one but several optimality criteria are set at once, it is possible to use an approach based on the Pareto principle.

**Figure 12:**
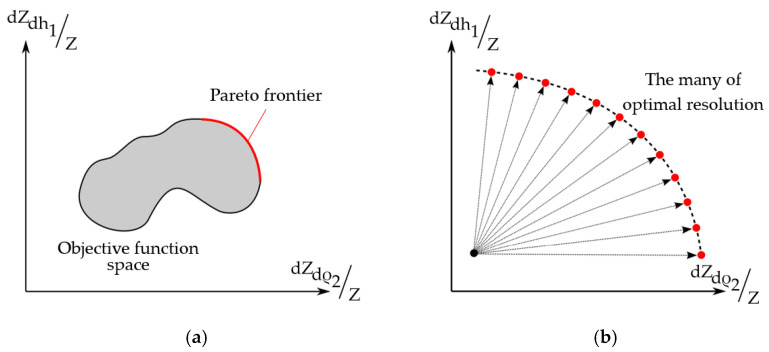
(**a**) Pareto frontier; (**b**) global multiobjective problem statement.

The Pareto optimal solution’s main idea is such a feasible solution that cannot be improved by any of the available criteria without worsening by some other. The solution to the multiobjective optimization problem is the Pareto set of all Pareto-optimal feasible points. Figure 12 shows the Pareto set corresponds to the Pareto frontier. In the Pareto set, points are not comparable with each other; i.e., all solutions to the problem are equivalent [44].

**Figure 13:**
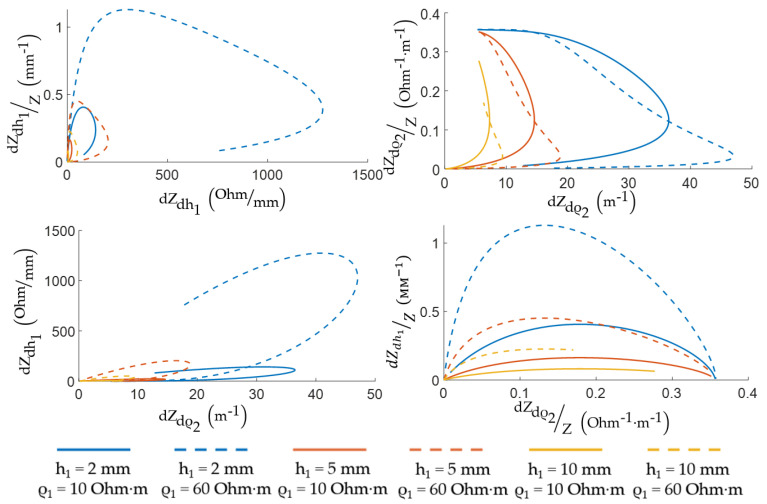
Pareto sets for EI sensitivity criteria.

In these studies, when constructing Pareto sets, the interelectrode distance a, which is varied in the range from 0 to 0.03 m, was used as a variable, and the EI sensitivity criteria were used as parametric functions. Figure 13 shows the results.

**Figure 14:**
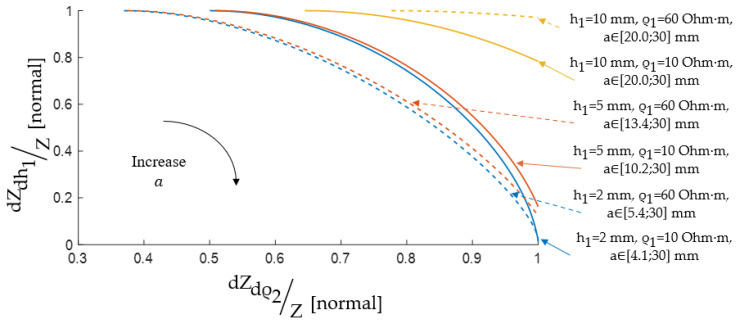
The Pareto optimal solutions are set for the interelectrode distance choice.

Figure 14 shows the results of determination of the ES size based on the Pareto frontier representation set by dimensionless Pareto optimal solutions according to the criteria ${\mathrm{dZ}}_{{\mathrm{dh}}_{1}}/Z$ and ${\mathrm{dZ}}_{{d\rho }_{2}}/Z,$ since these criteria to a greater extent reflect the specifics, in fact, EI measurements.

## 4. Results and Discussion

In order to quantify the absolute changes in electrical impedance when performing an action, it is necessary to add to the calculations changes in the thickness of the skin-fat layer and the resistivity of muscle tissue, corresponding to real values. At the same time, we assume that there is no change in the resistivity of the skin-fat layer.

**Figure 15:**
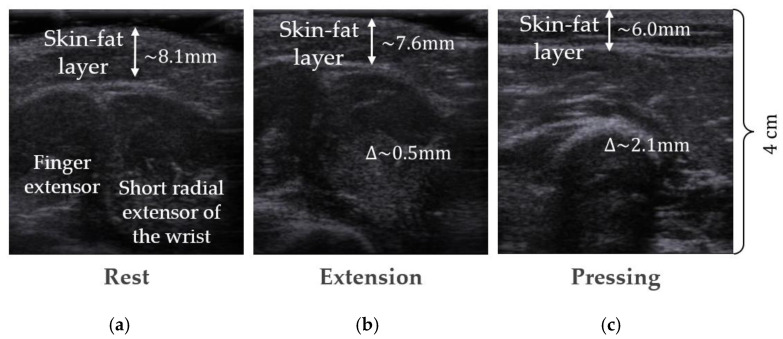
Example of transverse ultrasound images of internal structures of the forearm: (**a**) at rest; (**b**) when performing a wrist extension; (**c**) with an increase in the pressing force of the ultrasonic sensor to the skin surface.

The change in the thickness of the skin-fat layer was assessed based on the analysis of the features of the morphological changes in the forearm during the performance of actions carried out in the framework of the authors’ previous studies using ultrasound [45] and MRI [43]. Figure 15 shows that using the means for graphic assessment of physiological parameters according to the images obtained, the thinning of the skin-fat layer was determined upon exposure, on average, by 0.5 mm for different volunteers without pressing.

In the framework of previous studies by the authors of [45], the measured in vivo longitudinal electrical resistance of muscle tissue was comparable to the literature data obtained on the isolated tissues [8,18,19,20,34]. Measurements in the projection of the center of the muscle body show that the resistivity of the muscle in the longitudinal direction increases by an amount of the order of 5%, which is associated with muscle contraction.

Thus, using the analytical solution for the two-layer model (3) and the presented changes in the thickness of the skin-fat layer and the resistivity of the muscle tissue, the absolute values of the electrical impedance were calculated. These data are presented in Table 2 for electrode system sizes estimated using the Pareto optimality method described in the previous section. It is possible to trace how to change the contributions to the EI change for the two processes presented at ES different sizes. The table also shows the ratios $\Delta Z_{\Delta h_{1}}/\Delta Z_{\Delta \rho_{2}}\;$as EI changes due to these processes.

It is noted that it is necessary to use a larger ES to meet the criterion ${\mathrm{dZ}}_{{d\rho }_{2}}/Z$. The $a_{\min}$ value corresponds to the selected acceptable interelectrode distance based on the ${\mathrm{dZ}}_{{\mathrm{dh}}_{1}}/Z$ criterion, while the $a_{\max}$ value is based on the ${\mathrm{dZ}}_{{d\rho }_{2}}/Z$ criterion. In this case, the acceptable size selection is made based on the range of values from $a_{\min}$ to $a_{\max}$. When choosing the interelectrode distance, closer to $a_{\min}$, the ES will be more sensitive to changes in the skin-fat layer thickness; when closer to $a_{\max}$, it will be more sensitive to changes in muscle resistivity.

The approach analysis also shows that if the skin-fat layer conductivity is known and determined, for example, using the ES small size, its thickness and its change through ultrasound investigation, then the magnitude and change in the contracting muscle conductivity can be found as a result of the optimization procedure (8). The research development allows a more systematic approach to obtaining knowledge and data on the various muscle tissues during their contraction conductivity.

(8) $$\Delta \rho_{2}{=f}^{-1}\left({a,h}_{1}{,\rho }_{1},\Delta Z_{\Delta \rho_{2}}\right)$$

**Figure 16:**
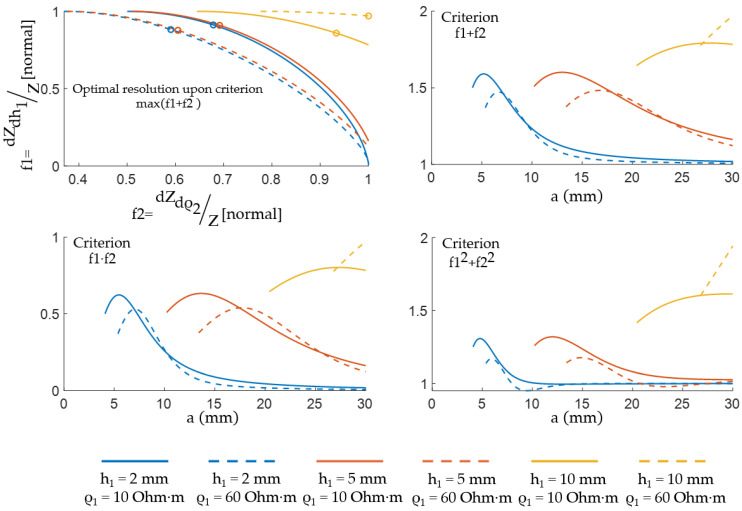
Multiobjective optimization problem solving for ${\mathrm{dZ}}_{{\mathrm{dh}}_{1}}/Z$ и ${\mathrm{dZ}}_{{d\rho }_{2}}/Z$.

Pareto optimal solutions generally contain an infinite number of points. The optimization is carried out using optimization algorithms, as a result, the problem of multiobjective optimization is reformulated so that it can be solved. For this reason, Figure 16 shows that the search for an acceptable ES size is limited to the case when the problem of maximizing the result of mathematical operations on objective functions—for example, sum, the sum of squares product—is considered.

The circles highlight the optimal solutions based on the objective functions’ sums corresponding to their maximum values. Table 3 shows the acceptable ES sizes for the cases considered. Furthermore, this approach can be used to determine the acceptable ES size for different parts of the body individually.

## 5. Conclusions

In this paper, a method for determining the acceptable ES size for forearm muscle activity EI measurements using the method for assessing Pareto optimality according to criteria considering the skin-fat layer individual parameters is proposed for the first time. The sensitivity criteria were the EI absolute and relative changes in the skin-fat layer thickness and the muscle tissues resistivity. These mechanisms are the basis for the EI myography signals conditioning. In this case, the skin-fat layer thickness and its change can be determined, for example, using ultrasound investigation, and the skin-fat layer conductivity using small ES sizes.

To achieve the study results, based on the ultrasound investigation, MRI, and data on the tissues’ electrophysical properties, analytical and EI signal conditioning numerical models during muscle contraction were built. For the EI mathematical description relationships, a forearm two-layer model was reasonably chosen, which was represented by the skin-fat layer and muscles, which adequately describes the change in EI when hand actioning, provided that the ES size does not exceed the characteristic dimensions of the limb. At the same time, to simplify the mathematical model, the electrodes themselves were represented by points, which is acceptable when analyzing electrical impedance changes, considering the previous studies of the authors [38].

Thus, the proposed mathematical model makes it possible to perform the calculations necessary to select the Pareto optimal sizes of the ES from the sensitivity of the EI measurements of muscle activity point of view, which were expressed by a change in the conductivity of muscle tissue and a change in the thickness of the skin-fat layer, considering individual parameters. These results can be used to develop systems for bionic control of the forearm based on EI. The study results allow considering the issues of solving inverse problems of limbs muscular activity electrical impedance probing in order to obtain new knowledge related to the muscle contraction biomechanical and morphological features and the EI myography signals conditioning mechanisms.

## Figures and Tables

**Table 1:** The error in simplifying the EI models in comparison with the anatomical.

	Single Cut		Ellipsoidal		Cylindrical		Planar 3 Layers		Planar 2 Layers	
a (mm)	S (Ohm)	S/Z	S (Ohm)	S/Z	S (Ohm)	S/Z	S (Ohm)	S/Z	S (Ohm)	S/Z
5	4.93	0.02	7.59	0.02	8.16	0.03	6.28	0.02	6.07	0.02
10	14.97	0.14	9.60	0.07	9.34	0.07	6.99	0.05	6.17	0.04
15	9.56	0.16	8.45	0.10	10.43	0.14	7.19	0.09	5.69	0.07
20	5.72	0.14	6.32	0.12	11.93	0.21	8.24	0.16	5.01	0.10

**Table 2:** Interelectrode distance selection at ∆ρ_2_ = 5%, ∆h_1_ = 0.5 mm.

Parameters		$\mathrm{Criterion}\;{\mathrm{dZ}}_{{\mathrm{dh}}_{1}}/Z$					$\mathrm{Criterion}\;{\mathrm{dZ}}_{{d\rho }_{2}}/Z\;(\mathrm{for}\;\mathrm{Threshold}\;0.7)$				
$\rho_{1}\;$ [Ohm*m]	$h_{1}\;[\mathrm{mm}]$	$a_{\min}\;[\mathrm{mm}]$	Z [Ohm]	$\Delta Z_{\Delta h_{1}}\;[\mathrm{Ohm}]$	$\Delta Z_{\Delta \rho_{2}}\;[\mathrm{Ohm}]$	$\Delta Z_{\Delta h_{1}}/\Delta Z_{\Delta \rho_{2}}$	$a_{\max}\;[\mathrm{mm}]$	Z [Ohm]	$\Delta Z_{\Delta h_{1}}\;[\mathrm{Ohm}]$	$\Delta Z_{\Delta \rho_{2}}\;[\mathrm{Ohm}]$	$\Delta Z_{\Delta h_{1}}/\Delta Z_{\Delta \rho_{2}}$
10	2	4.0	203.7	41.2	5.0	8.3	5.3	123.2	20.0	4.3	4.7
10	5	10.1	80.0	6.5	2.0	3.3	13.3	49.0	3.4	1.7	2.0
10	10	20.0	40.7	1.7	1.0	1.7	20.0	40.7	1.7	1.0	1.7
60	2	5.3	277.3	126.9	5.0	25.4	7.3	103.0	30.4	3.5	8.6
60	5	13.4	106.9	22.5	2.0	11.4	18.4	40.3	6.0	1.4	4.2
60	10	20.0	134.4	13.1	1.2	10.7	20.0	134.4	13.1	1.2	10.7

**Table 3:** Acceptable ES sizes for multiobjective optimization.

Parameters		$a_{\mathrm{opt}}\;[\mathrm{mm}]$		
$\rho_{1}\;$ [Ohm·m]	$h_{1}\;\left[\mathrm{mm}\right]$	$f_{1}{+f}_{2}$	$f_{1}\cdot f_{2}$	$f_{1}{}^{2}{+f}_{2}{}^{2}$
10	2	5.2	5.5	4.8
10	5	13.0	13.6	12.0
10	10	27.7	27.3	29.9
60	2	6.7	7.1	5.9
60	5	16.8	17.7	14.9
60	10	30.0	30.0	30.0

**Figure A1:**
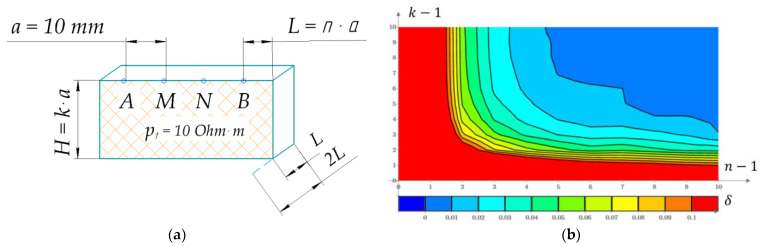
EI model (**a**) and the results of comparison EI simulated with the analytical one (**b**) to determine the geometric dimensions.

**Figure A2:**
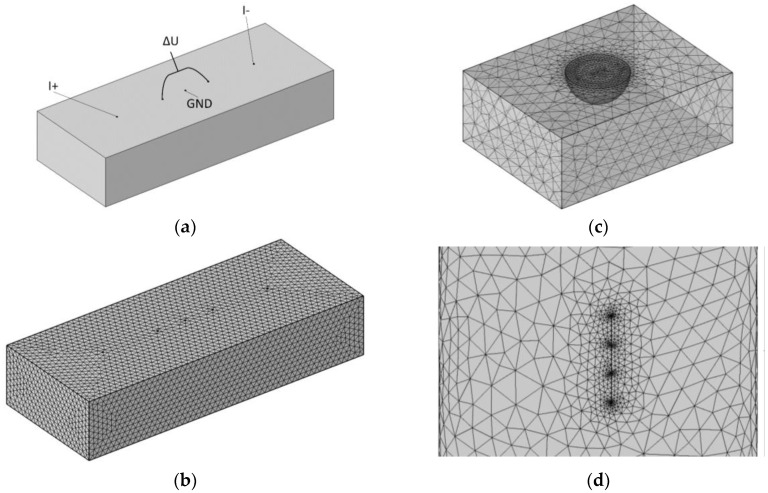
Various dividing models for the computational grid: (**a**) solid model; (**b**) global division; (**c**) local refinement; (**d**) uniformly increasing division.

**Figure A3:**
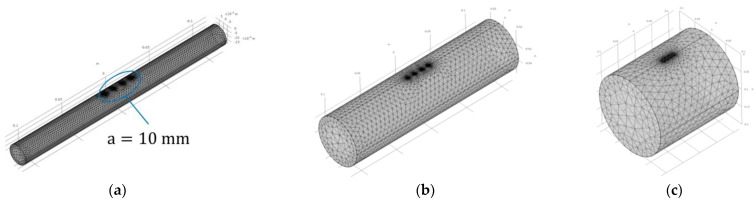
Cylindrical models with different ratios of radius to interelectrode distance: (**a**) R/a = 1, (**b**) R/a = 3, (**c**) R/a = 10.

**Figure A4:**
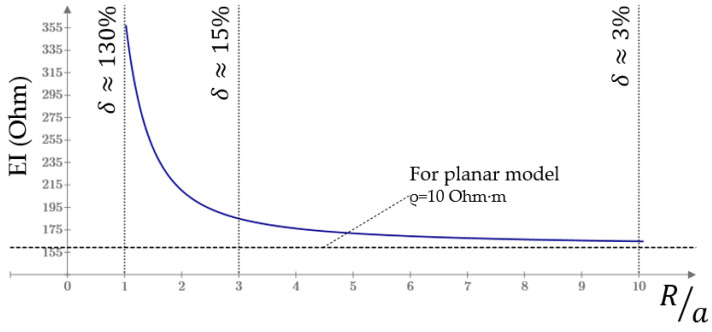
Simplification of the homogeneous cylindrical model to a planar one (R is the cylindrical model radius; a is the interelectrode distance).

## Data Availability

The data presented in this study are available on request from the corresponding author.

## Appendix A. EI Model Acceptable Size Determination

There is a known method for finding the homogeneous infinite medium resistivity based on a mathematical model in which CE (A, B) and PE (M, N) are assumed to be point-like. With this problem statement, the medium resistivity ρ is calculated by (A1), where K is the coefficient that determines the installation geometric parameters (the distance between the electrodes), ∆U is the potential difference across the PE, and I is the current flowing through the CE.

(A1) $$\rho =K\frac{\Delta U}{I}$$

Then, the analytical solution for finding the value of EI Z can be written as (A2).

(A2) $$Z=\frac{\rho }{K}$$

The EI analytical value in the case of the location of the electrodes by the Wenner tetrapolar system (with an equidistant distance between the electrodes) has the form (A3).

(A3) $$Z=\frac{\rho }{2\pi \alpha }$$

The simulation based on the EI calculation and its subsequent comparison with the analytical value was carried out to determine the model size, which makes it possible not to take into account the effects of the finite size and to consider it conditionally semi-infinite. Figure A1 shows the model was represented as a semi-infinite homogeneous medium with a specific electrical resistance ρ = 10 Ohm·m, which was close to the skin-fat layer resistance at a frequency of 100 kHz. All model geometrical dimensions are specified concerning the interelectrode distance a value, taken equal to 0.01 m, to move away from the absolute dimensions.

Based on the finite element simulation results, the EI simulated values deviation that was obtained with the global mesh partitioning “Extremely Fine” standard parameters, with the model parameters’ length and width L ≥ 8a, depth H ≥ 8a from the analytical solution (A3) is no more than 1% (relative error δ ≤ 0.01). This error confirms the reliability of the obtained simulation results, and the corresponding dimensions in the study calculations were used.

## Appendix B. Computational Model Grid Type Dividing Substantiation

One of the steps in building a computational model in COMSOL Multiphysics is choosing a mesh for the model, on which the numerical computation accuracy depends. Figure A2 shows three types of dividing were considered: global division, local refinement, and uniformly increasing division to unify the dividing principle to be able to compare the results of calculating the EI different models.

Based on the finite element simulation results using a method similar to that presented in Appendix A, it has been determined that the last case of mesh division- uniformly increasing division is the most efficient one. This division with the parameters presented in Table A1 allows obtaining the EI simulation results with a minimum deviation from the analytical value as well as increasing the performance of the calculation due to the smaller number of model elements. These parameters were used in the study calculations.

**Table A1:** Mesh building options when dividing models.

Elements’ Parameters		Elements Distribution	
Maximum size	10 mm	Quantity	10
Minimum size	0.01 mm	Ratio	10
Rate of increase	1.4	Ascending formula	Arithmetic
Curve factor	0.4	Symmetric distribution	Yes
Narrow regions resolution	0.7		
Element type	Tetrahedron		

## Appendix C. Computational Model Type of Grid Division Substantiation

Figure A3 shows the part of the cylindrical model simplification to a planar one; finite element simulation was performed using a cylinder model. The radius increased while maintaining the interelectrode distance. With an increase in the ratio of the interelectrode distance to the cylinder radius, the EI value decreases, which at large values will converge with the analytical expression results (Figure A3). Thus, the EI planar models error in the case of using a small ES is minimal.

The use of models for which the limb characteristic radius is three times greater than the interelectrode distance makes it possible to approximate a cylindrical model with a single-layer planar model with an error in the EI value of no more than 15%. This is also taking into account the results simplifying models, which justifies the planar two-layer model choice to determine the skin thickness effect and the specific muscles resistance, as well as their chances in the action process, by the measured impedance value. Figure A4 shows the investigation results.
